# Nucleolin Is Required for DNA Methylation State and the Expression of rRNA Gene Variants in *Arabidopsis thaliana*

**DOI:** 10.1371/journal.pgen.1001225

**Published:** 2010-11-24

**Authors:** Frédéric Pontvianne, Mohamed Abou-Ellail, Julien Douet, Pascale Comella, Isabel Matia, Chinmayi Chandrasekhara, Anne DeBures, Todd Blevins, Richard Cooke, Francisco J. Medina, Sylvette Tourmente, Craig S. Pikaard, Julio Sáez-Vásquez

**Affiliations:** 1 UMR 5096 CNRS-IRD-University de Perpignan, Perpignan, France; 2 Department of Biology and Department of Molecular and Cellular Biochemistry, Indiana University, Bloomington, Indiana, United States of America; 3 UMR CNRS 6247, INSERM U931, University Blaise Pascal, Aubière, France; 4 Centro de Investigaciones Biológicas, Consejo Superior de Investigacion Científicas, Madrid, Spain; Stanford University, United States of America

## Abstract

In eukaryotes, 45S rRNA genes are arranged in tandem arrays in copy numbers ranging from several hundred to several thousand in plants. Although it is clear that not all copies are transcribed under normal growth conditions, the molecular basis controlling the expression of specific sets of rRNA genes remains unclear. Here, we report four major rRNA gene variants in *Arabidopsis thaliana*. Interestingly, while transcription of one of these rRNA variants is induced, the others are either repressed or remain unaltered in *A. thaliana* plants with a disrupted nucleolin-like protein gene (*Atnuc-L1*). Remarkably, the most highly represented rRNA gene variant, which is inactive in WT plants, is reactivated in *Atnuc-L1* mutants. We show that accumulated pre–rRNAs originate from RNA Pol I transcription and are processed accurately. Moreover, we show that disruption of the *AtNUC-L1* gene induces loss of symmetrical DNA methylation without affecting histone epigenetic marks at rRNA genes. Collectively, these data reveal a novel mechanism for rRNA gene transcriptional regulation in which the nucleolin protein plays a major role in controlling active and repressed rRNA gene variants in *Arabidopsis*.

## Author Summary

Chromatin remodeling plays a central role in controlling gene expression in all eukaryotic organisms. Chromatin can be found in a repressive or transcriptionally inactive state (heterochromatin) or in a more permissive or transcriptionally active state (euchromatin). The building block of chromatin is the nucleosome, which consists of four histones, H2A, H2B, H3, and H4, surrounded by 147 base pairs of DNA. In addition, a linker histone H1 directs the path of DNA between adjacent nucleosomes to form the chromatin fiber. Chromatin compaction also depends on DNA methylation and on a number of histone modifications, including methylation and acetylation of histone tails. However, other non-histone proteins are required to direct chromatin structure and remodeling. Nucleolin is a major nucleolar protein involved not only in rRNA transcription and processing of 45S pre–rRNA transcribed by RNA Pol I, but also in the control of RNA pol II transcription in the nucleoplasm. Through genetic, molecular, and immunocytological approaches, we studied the role of this protein *in vivo* in controlling rRNA chromatin structure and the expression of hundreds of clustered rRNA genes using the model plant *Arabidopsis thaliana*.

## Introduction

**Figure 1:**
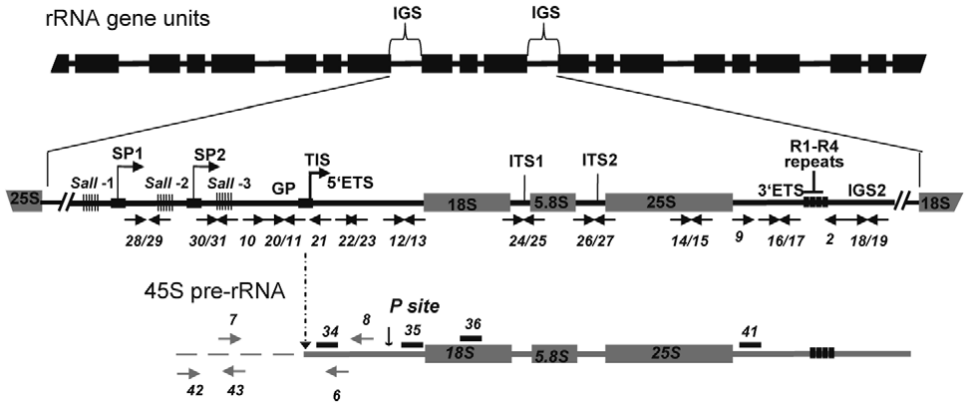
Representation of rRNA gene repeats transcribed by RNA polymerase I. The top portion shows tandemly-arrayed 18S, 5.8S and 25S rRNA genes separated by intergenic sequences (IGS). In the middle, an enlarged rRNA unit is presented, with positions of the gene promoter (GP) and two spacer promoters (SP1 and SP2) located between the three repeat elements containing Sal I restriction site DNA repeat elements (SalI-1, -2 and -3 repeats). The arrow in GP indicates the Transcription Initiation Site (TIS). The lower scheme represents the primary 45S pre-rRNA, containing the external transcribed spacers (5′ETS and 3′ETS), and the structural rRNA sequences (18S, 5.8S and 25S rRNA in gray boxes) separated by internal transcribed spacers (ITS1 and ITS2). Four repeat sequences located in the 3′ETS are represented (R1-R4). The vertical arrow shows the primary cleavage site (P) in the 5′ETS. Positions of primers used to amplify or detect rRNA gene and/or pre-rRNA sequences are shown.

In eukaryotic cells, ribosomal RNA genes (rRNA) are arranged in head-to-tail tandem arrays (depicted in Figure 1). The rRNA genes clustered at a single locus comprise nucleolar organizer regions (NORs), so named because the nucleolus, the site of ribosome synthesis, is organized around active rRNA genes during interphase [1]–[3]. Each rRNA gene transcription unit consists of sequences encoding a precursor transcript that includes the structural rRNAs (18S, 5.8S, 25S), the Internal Transcribed Spacers (ITS) and the External Transcribed Spacers (ETS). The rRNA gene units are separated from the adjacent gene in the array by an intergenic spacer (IGS) [4]. In plants, as in animals, coding sequences for the three structural rRNAs are highly conserved, even between distantly-related species, but the IGS and sequences which are removed during processing, including the ITS and ETS, are much less well conserved [5].

Analysis of complete IGS sequences reveals considerable length and sequence heterogeneity in different plant species, including radish, wheat, *A. thaliana*, *Brassica*, *Nicotiana* and *Solanum* species [6]. However, all ribosomal IGS contain repeated sequences. IGS organization in plant rRNA genes resembles IGS organization in most higher eukaryotes, including at least one array of tandemly-repeated sequences located upstream from the transcription initiation site (TIS). In *Xenopus* [7] and mouse [8], repeated sequences in this location have been shown to possess enhancer activity, increasing the expression of the adjacent promoter after injection into frog oocytes or embryos or after transfection into cultured cells. Though *Arabidopsis* spacer repeats cloned next to a *Xenopu*s rRNA gene promoter act as enhancers in frog oocytes [9], there is no evidence that they have analogous enhancer activity in plant cells [10]. In plants and animals not all rRNA genes are active and the mechanisms responsible for the selective activation and/or silencing of subsets of these genes have been sought for many years. Thus, questions persist as to whether or not IGS heterogeneity affects rRNA transcription; possibly playing a role in activation or silencing of rRNA genes or in stimulating rRNA transcription levels among active rRNA genes.

In the context of nucleolar dominance, activation or repression of specific rRNA genes is controlled mainly by rRNA chromatin modification [11]. In *Arabidopsis suecica*, an allotetraploid derived from *A. thaliana* and *A. arenosa*, the *thaliana* derived rRNA genes are repressed and those of *arenosa* are expressed [12]. The *thaliana* derived rRNA genes are hyper-methylated and are associated with histone H3 dimethylated on lysine 9 (H3^dimethyl^K9), whereas the *arenosa*-derived genes are hypo-methylated and are associated with histone H3 trimethylated on lysine 4 (H3^trimethyl^K4) [13]. Blocking *de novo* DNA methylation causes changes in histone modifications, and blocking histone deacetylation induces cytosine demethylation. Indeed, knockdown by RNAi of *HDA6* and *HDT1* (histone deacetylase and histone deacetylase –like protein encoding genes respectively) in *A. suecica* causes the derepression of *thaliana*-derived rRNA genes [13]–[14]. An RNAi knockdown screen in *Arabidopsis* identified DRM2, a *de novo* cytosine methyltransferase, and MBD6 and MBD10, two methyl cytosine binding domain proteins, as activities also required for nucleolar dominance [15]. More recently, it was demonstrated that HDA6 plays a role in activation of specific rRNA genes in *A. thaliana* [16].

Chromatin decondensation is generally correlated with transcriptional gene activation, in such a way that the amount of decondensed chromatin reflects gene expression activity, either real or potential [17]. *AtHDA6* and *AtNUC-L1* (a NUCleolin-Like protein), play a role in rRNA chromatin condensation in *Arabidopsis* [14], [18]. Decondensation of rRNA chromatin has no discernible effect on rRNA mature transcript levels in *AtHDA6* mutants [14]. However, in plants with a disrupted *AtNUC-L1* gene, the level of transcripts initiated from the gene promoter decreases and pre-rRNA cleaved at the P site accumulates [18], indicating that AtNUC-L1 plays a major role in controlling homeostatic rRNA gene expression.

In eukaryotic cells, nucleolin is one of the most abundant non-ribosomal proteins in the nucleolus [19]. Studies *in vitro* [20]–[22] or in animal cells with reduced amounts of nucleolin [23], [24], clearly show that nucleolin plays a role in different steps in ribosome biogenesis, including RNA pol I transcription and processing of pre-rRNA. However, a role for nucleolin in mechanisms responsible for controlling activation and/or silencing of specific rRNA genes has not been investigated.

Here we present evidence for a role of AtNUC-L1 in controlling activation and repression of a specific subset of rRNA genes located in distinctive NORs. Our findings indicate that AtNUC-L1 might play a central role in a mechanism responsible for switching expression of rRNA genes that involves IGS transcription and symmetric DNA methylation.

## Results

### Specific rRNA variants are expressed and/or repressed in *Atnuc-L1* plants

We previously reported that disruption of a nucleolin like protein gene from *A. thaliana* (*AtNUC-L1*) affects nucleolar structure, rRNA chromatin condensation and accumulation of pre-rRNA [18]. To determine if *AtNUC-L1* gene disruption affects transcription activation and/or repression of specific rRNA genes in *Atnuc-L1* plants, we carried out a systematic cloning and sequencing strategy to characterize 3′ETS rRNA genes and pre-rRNA transcribed sequences from WT and two *Atnuc-L1* mutant lines.

**Figure 2:**
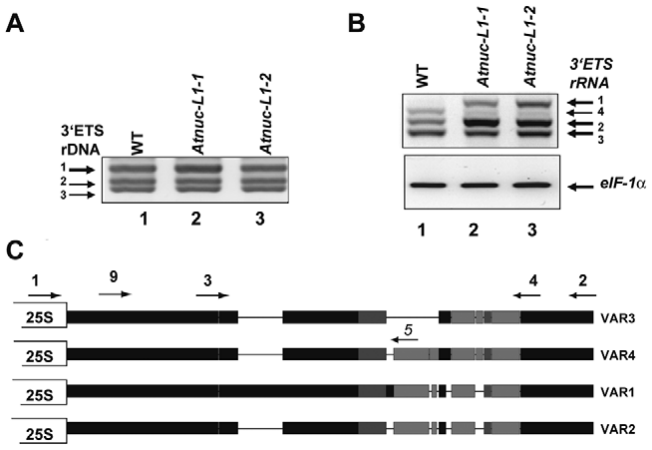
*A. thaliana* encodes and expresses specific rRNA gene variants. A) PCR amplification of 3′ETS rRNA gene sequences using primers p1/p2 and genomic DNA from WT (lane 1) and Atnuc-L1 mutant plants (lanes 2 and 3). Arrows show 3′ETS rRNA genes VAR1, VAR2 and VAR3 based on expected sizes and sequencing data (Figure S3). B) One Step RT-PCR analysis using primers p3/p4 to detect 3′ETS pre-rRNA sequence variants in WT (lane 1) and Atnuc-L1 mutant (lanes 2 and 3) plants. C) Representation of 3′ETS rRNA gene variants. Black, dark and/or light grey rectangles represent high, medium or low sequence identity respectively. Lines joining the rectangles indicate deletions in sequences of each rRNA gene variant. Positions of primers p1-p5 and p9are indicated.

As shown in Figure 2A, PCR reactions with primers *p1/p2* (shown in Figure 2C) to amplify 3′ETS rRNA gene sequences detect three major bands in genomic DNA prepared from WT (lane 1) and *Atnuc-L1-1* and *Atnuc-L1-2* plants (lanes 2 and 3). Cloning and sequencing analysis indicates that the genome of *A. thaliana* contains at least three major rRNA gene variants, *VAR1*, *VAR2* and *VAR 3*. Sequencing analysis of ∼90 3′ETS rRNA gene clones showed that 48% of the sequences correspond to *VAR1*, while 30% and 22% correspond to *VAR2* and *VAR3* respectively. An additional rRNA gene variant (*VAR4*) was also identified by RT-PCR (see below) and confirmed by PCR amplification of genomic DNA using primers specific to this novel low copy number variant (data not shown). These variants can be distinguished from each other by the presence of a large insertion (*VAR1*) or deletions (*VAR3*) and/or additional short insertions or deletions (*VAR2* and *VAR4*) located in the 3′ETS rRNA gene region (Figure 2C and Figure S3). Furthermore *VAR2* and *VAR3* can be separated into two sub variants, *VAR2*, *VAR2a* and *VAR3*, *VAR3a*, based on a short three base pair deletion (CAC and CGC respectively, double over lined in Figure S3). *VAR1* contains four repeat sequences (R1-R4) located downstream from the 3′ETS cleavage site, in contrast to *VAR2*, *VAR3* and *VAR4* which contain only two complete repeats (R1 and R4). In contrast, PCR amplification of rRNA gene sequences (from −520 to +209) and sequencing analysis of ∼50 rRNA clones did not reveal major sequence variations in the promoter and/or in the 5′ETS region (data not shown).

In order to determine if some or all of these rRNA gene variants are expressed, we performed RT-PCR analysis using primers *p3/p4* that amplify shorter fragments than those used in Figure 2A, allowing better resolution between the four bands. As shown in Figure 2B, the amplification band corresponding to *VAR1* (greater size) accumulates preferentially in *Atnuc-L1* mutant plants (lane 2 and 3). No amplification band corresponding to VAR1 was detected in WT plants (lane 1). Amplification bands corresponding to *VAR2* and *VAR4* accumulate or decrease respectively in the *Atnuc-L1* mutant compared to amplification signals in WT plants. In contrast, no significant differences in the amplification signal corresponding to *VAR3* was observed between WT and *Atnuc-L1* plants. The identity of each band was confirmed by sequencing. Similar results were obtained by RT-PCR reactions using the primers *p1* and *p2* used for amplification of rRNA genomic sequences (data not shown). Lower accumulation of rRNA *VAR1* in *Atnuc-L1*-*1* compared to *Atnuc-L1-*2 is not due to variations in the expression of this gene in individual plants. Indeed, RT-PCR analysis revealed similar increased amount of rRNA *VAR1* in all individual *Atnuc-L1-1* plants analyzed compared to wild-type (Figure S7).

To verify that activation of rRNA gene *VAR1* is associated with the absence of AtNUC-L1 protein, we transformed *Atnuc-L1* mutant plants with AtNUC-L1 genomic sequences under the control of its own promoter (Figure S2). Complementation of *Atnuc-L1* mutants with AtNUC-L1 genomic sequences restore, at least partially, the amount of rRNA variants to those observed in WT plants (Figure S2B). Partial complementation is probably due to the fact that in these plants levels of AtNUC-L1 are significantly lower than those observed in WT (Figure S2C). This might suggest a dosage effect of AtNUC-L1 in rRNA gene variant expression. However, expression of transgenic AtNUC-L1 is sufficient to restore plant growth and developmental defects of *Atnuc-L1* mutant plants (Figure S2A).

In conclusion, these analyses reveal the existence of several variants of rRNA genes, including an rRNA gene variant (*VAR1*), silent in wild-type plants, which is derepressed in *A. thaliana* plants lacking the AtNUC-L1 protein. Overall, they demonstrate a striking balance between active and inactive rRNA gene variants in WT and *Atnuc-L1* plants.

### Expression of rRNA gene variants in *A. thaliana* wild-type plants

Nucleolin like protein gene expression is up or down -regulated in meristematic tissues and at specific stages of plant development [18],[25]–[28]. In order to determine if the rRNA gene variants (*VAR1-4*) are differentially expressed in WT plants, we performed One Step RT-PCR using primers *p3/p4* (shown in Figure 2C) to amplify 3′ETS pre-rRNA sequences in different organs and or plant conditions.

**Figure 3:**
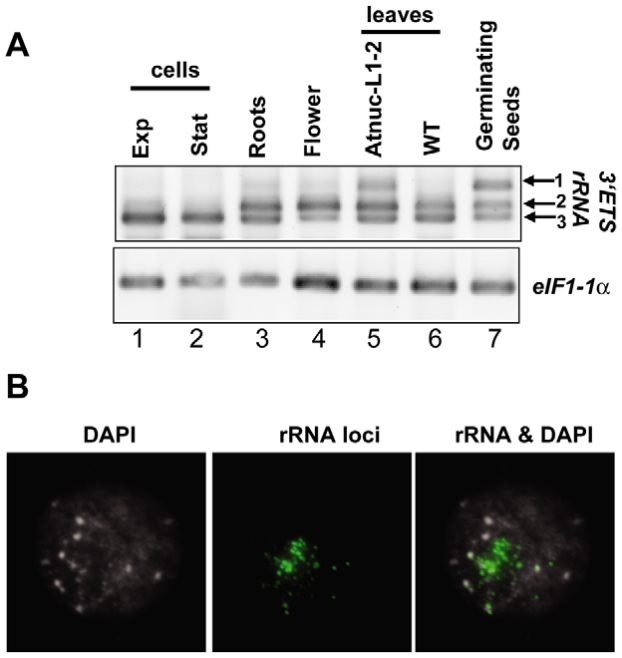
rRNA gene variants are differentially expressed in *A. thaliana* plants. A) One Step RT-PCR analysis was using primers *p3/p4* to detect 3′ETS pre-rRNA sequence variants in exponentially (lane 1) or stationary growing (lane 2) cells; in roots (lane 3), flower (lane 4), leaves from *Atnuc-L1-2* mutant (lane 5) or WT (lane 6) plants or 2 days germinating seeds (lane 7). RNA from roots and leaves were prepared from 15 days old WT and *Atnuc-L1* plant grown in MS medium as described previously [18]. Panel eIF1α; amplification of eIF1α mRNA was performed to control PCR reaction and total RNA in different samples. B) NOR condensation is affected in 2 day germinating seeds. Left, chromatin counterstained with DAPI, Middle FISH using 25S rRNA probe and Right, superposition of FISH and DAPI images.

As observed in Figure 3A, the 3′ETS rRNA *VAR2* and *VAR3* are detected in roots, flowers, leaves and germinating seeds (lanes 3, 4 and 6, 7). rRNA *VAR1* is detected essentially in seeds imbibed for 48 hrs (lane 7), though a very weak band is amplified in roots and flowers (lanes 3 and 4). In exponential or stationary cell culture (lanes 1 and 2), the rRNA *VAR3* corresponds to the major amplification band. As shown previously, *VAR1* is also detected in *Atnuc-L1* mutant plants, used here as a control (lane 5). PCR reactions without reverse transcriptase did not reveal amplification products in any of the samples tested (data not shown). Amplification of *eIF1α* was performed to control RNA amounts in each sample (panel eIF1α).

In a context of nucleolar dominance, at 2 days post germination in the allelotetraploid *Arabidopsis suecica* the *thaliana* -derived NORs are decondensed and the rRNA genes are expressed [29]. Here, to verify the chromatin state in the 2-days germinating *A. thaliana* seedlings expressing the rRNA *VAR1* gene (refer to Figure 3A), we performed FISH analysis. As shown in Figure 3B, in the nucleus of 2 days germinating seedlings the rRNA chromatin is immature. In these nuclei several FISH signals are observed using a 25S probe, but these are smaller compared with those observed in 15 –day old plants (see Figure 6B). This is indicative of decondensed fibers of active rRNA genes, which can not be observed because are too thin, juxtaposed with condensed portions of heterochromatic rRNA signals (green labeling).

From these results, we conclude that rRNA variants are developmentally regulated in *A. thaliana* plants and chromatin state plays a major role in controlling the expression of rRNA VAR1.

### 45S pre–rRNA precursors are accurately processed in *Atnuc-L1* plants

**Figure 4:**
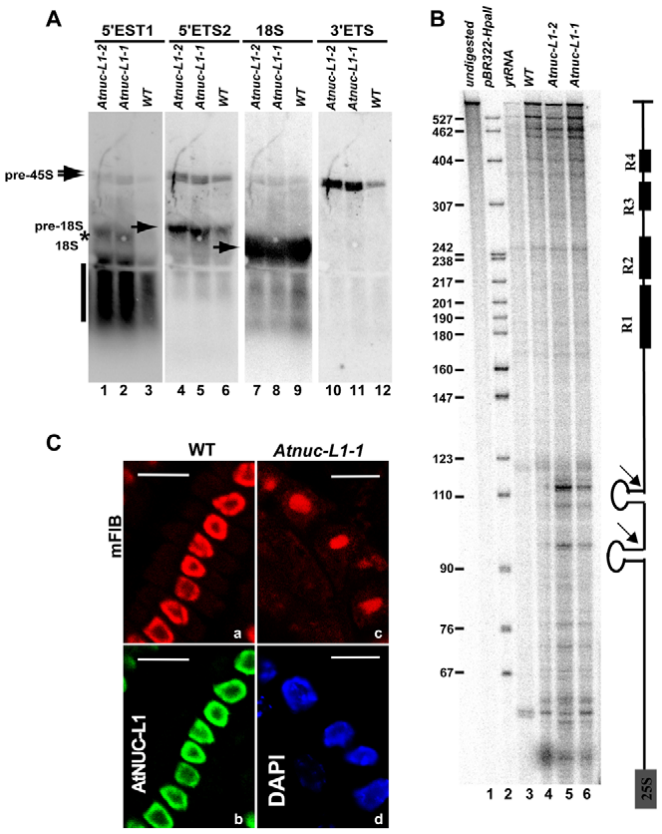
Processing of accumulated pre-rRNA in *Atnuc-L1* mutant plants is accurate. A) Northern blot analysis using total RNA isolated from WT and Atnuc-L1-1 mutant plants and [γ^32P^] 5′-end labeled primers p34, p35, p36 and p41 to detect 5′ETS1 (lanes 1–3), 5′ETS2 (lanes 4–6), 18S (lanes 7–9) and 3′ETS (lanes 10–12) pre-rRNA sequences respectively. The asterisk and vertical bar indicate expected 5′ETS cleave off and exonucleolityc products (See also Figure S4). B) RNAseA/T1 protection analysis was carried out with a radiolabelled probe complementary to the 3′ETS (right). The assay was performed with total RNA from WT (lane 4) and Atnuc-L1 (lanes 5 and 6) or with yeast tRNA as a control (lane 3). A control lane loaded with undigested riboprobe is shown (lane 1). Lane 2, pBR322 digested with HpaII and 5′end labeled with T4 PNK and [γ^32P^] ATP. C) Immunolocalization of fibrillarin in roots from WT and Atnuc-L1-1. Panel mFIB; Fibrillarin appears more abundant in the nucleolus of WT (a) than in the disorganized nucleolus of Atnuc-L1 plants (c) [18]. The nucleolar localization of fibrillarin practically overlaps the localization of AtNUC-L1 (b). Fibrillarin was detected with antibodies against mouse fibrillarin (mFIB 72B9) and Alexa-546 and AtNUC-L1 with antibodies against peptide AtNUC-L1 and Alexa-488. Chromatin in Atnuc-L1-1 is counterstained with DAPI (d). Bar, 10 µm.

We previously reported that pre-rRNA cleaved at the primary cleavage site in the 5′ETS region (P site) accumulates in *Atnuc-L1-1* mutant plants [18]. Here we examine accumulation of 45S pre-rRNA and processing in the 3′ETS region in *Atnuc-L1-1* plants because 3′ETS cleavage is a co-transcriptional event that releases 45S pre-rRNA precursor [6]. To verify accumulation of these precursors in *Atnuc-L1-1* plants we performed northern blot analysis using oligonucleotide probes *p34* and *p35* that specifically match 5′ETS sequences located either upstream and downstream from the P cleavage site, respectively, and oligonucleotide *p41* to detects 3′ETS region (position shown in Figure 1 and Figure S4). As observed in Figure 4A, hybridization using *p34, p35* and *p41* primers generates stronger radioactive signals in the *Atnuc-L1* mutant (lanes 1–2, 4–5 and 10–11) than in WT samples (lanes 3, 6 and 12). The larger radioactive signals detected using *p41* (lanes 10–12), but also by *p34* and *p35* (lanes 1–6), correspond to the 45S pre-rRNA. The signals detected only by primer *p35* correspond to an intermediate of 18S precursor forms (lanes 4–6). The smear detected with primer *p34* might be due to exonucleolityc degradation of a 5′ETS cleave off product, while the signal indicated by an asterisk might correspond to a 5′ETS product produced by an alternative cleavage event upstream from the P site (lanes 1–3). Hybridization using primer *p36* detects similar amounts of 18S rRNA in WT and *Atnuc-L1* samples (lanes 7–9), Accumulation of pre-rRNA in *Atnuc-L1-1* mutants was also observed by Northern blot using primers specific to ITS1, ITS2, 5.8S and 25S sequences. As for 18S we did not observe accumulation of either mature 5.8 or 25S rRNA (Figure S4).

To determine if the 45S pre-rRNA is accurately processed at the 3′ETS cleavage site, we performed an RNase protection assay (Figure 4B). This analysis revealed two major radioactive signals of ∼95 and ∼110 nucleotides that accumulate to higher levels in *Atnuc-L1* (lanes 5 and 6) versus WT (lane 4) plants, as expected. These signals map to cleavage sites in the stem base of two hairpin-loop structures that may be recognized by a RNase III implicated in co-transcriptional cleavage of pre-rRNA [30], [31]. The mapped cleavage sites are located upstream from four repeat sequence elements named R1 to R4 (Figure S3). Slight variations were observed upstream from the major cleavage site signals when the pattern of radioactive signal was compared between WT and *Atnuc-L1* samples.

In *Atnuc-L1* plants, the nucleolus is disorganized and this might affect the nucleolar localization of factors involved in the nucleolar step of rRNA synthesis [18]. Fibrillarin is a major nucleolar protein factor required for primary cleavage in the 5′ETS and methylation of pre-rRNA [32]. As shown in Figure 4C, after incubation of *A. thaliana* meristematic cells with murine monoclonal anti-fibrillarin autoantibody (mFIB, 72B9), the immunofluorescent labeling seems specifically concentrated in the nucleolus of both WT (a) and *Atnuc-L1-1* plants (c). In WT plants the nucleolus is immunostained with antibodies against AtNUC-L1 (b) and in *Atnuc-L1* mutants, DAPI is observed as a ring around the dark and unstained nucleolus (d).

Taken together, these results indicate that accumulated 45S pre-rRNA in *Atnuc-L1* plants is accurately processed and suggest that pre-rRNA processing activity in the nucleolus is essentially not affected in the absence of the AtNUC-L1 protein.

### RNA pol I transcription from the IGS in *Atnuc-L1* mutant plants

Accumulation of pre-rRNA, which is higher in mutant compared to WT plants, can be controlled by increasing the number of rRNA genes being transcribed and/or by increasing the number of actively transcribing RNA Pol I enzymes [33], [34]. To obtain insight into the transcriptional origin of pre-rRNA transcripts accumulated *in Atnuc-L1* plants, we used Chromatin Immuno Precipitation (ChIP) and Reverse Transcriptase-Polymerase Chain Reaction (RT-PCR) experimental approaches.

**Figure 5:**
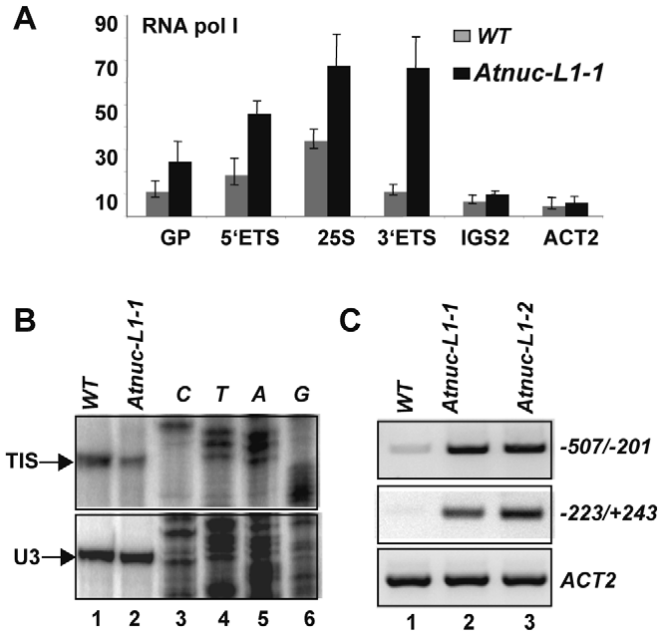
Accumulation of RNA polymerase I and rRNA transcripts from the IGS in *Atnuc-L1* plants. A) Chromatin samples prepared from WT (grey bars) and Atnuc-L1-1 mutant (black bars) plants were immunoprecipitated with antibodies against the largest RNA pol I subunit. ChIP samples were amplified with specific primers to GP (p10/p11), 5′ETS (p12/p13), 25S (p14/p15), 3′ETS (p16/p17) and IGS2 (p18/p19) rRNA sequences. Amplification of the ACT2 gene was performed to control ChiP and qRT-PCR reactions. B) Primer extension was performed using total RNA extracted from WT (lane 1) and AtnucL-1 (lane 2) plants and primer p6 for mapping transcription initiation site (TIS) from the gene promoter (GP). Primer extension using primer 3AtU3 that accurately maps the 5′ end of U3snoRNA was used to control similar amounts of total RNA in each reaction. Lanes 3–6 show DNA sequencing reactions used to map accurately the transcription initiation site (panel TIS) or verify the expected size for U3 snoRNA amplification. C) RT-PCR analysis using primers p42/p43 and p7/p8 to detect transcription from IGS(from −507 to −201 and from −223 to +243 respectively). One Step RT-PCR reaction of ACT2 transcripts was performed to control similar amount of total RNA in WT (lane 1) and Atnuc-L1 (lanes 2 and 3) samples.

To compare the level of RNA Pol I associated with rRNA genes in WT and *Atnuc-L1* plants we performed ChIP using antibodies against the largest RNA pol I subunit and specific primers to amplify Gene Promoter (GP, *p10/p11*), 5′External Transcribed Spacer (5′ETS, *p12/p13*), 25S rRNA (25S, *p14/p15*), 3′External Transcribed Spacer (3′ETS, *p16/p17*) and Internal Gene Spacer 2 (IGS2, *p18/*p19) sequences (Figure 5A). Quantitative PCR analysis revealed higher *GP*, *5′ETS*, *25S* and *3′ETS* amplification signals in samples from *Atnuc-L1-1* plants (black bars) compared to signals in samples from WT plants (grey bars). In contrast, when the region located just downstream from the four repeat sequences (IGS2) was analyzed, the PCR amplification signals in samples from *Atnuc-L1* and WT plants were similar.

On the other hand, primer extension analysis shown in Figure 5B reveals that the level of transcripts initiated at the Transcription Initiation Site (TIS) in the gene promoter are lower in *Atnuc-L1* than in WT plants. Accordingly, we would expect that accumulated pre-rRNA transcripts in *Atnuc-L1* plants are generated from alternative transcription initiation sequences, possibly from spacer promoters (SP) located in the IGS (Figure 1). Indeed, it has been shown that RNA pol I is capable of initiating transcription from spacer promoter rRNA sequences in *A. thaliana* WT plants [9]. We performed RT-PCR reactions to verify transcription of pre-rRNA from the IGS in *Atnuc-L1* mutant plants. As shown in Figure 5C, when PCR reactions were performed with oligonucleotides *p42/p43* and *p7/p8* to amplify sequences from −507 to −201 and from −223 to +243 respectively (shown in Figure 1) a specific band was amplified to a higher level in RNA samples from *Atnuc-L1* mutants (lanes 2 and 3) compared with WT (lane 1) plants. RT-PCR reactions using primers that specifically amplify *AtACT2* sequences were performed to verify similar amounts of RNA in each reaction (Panels *ACT2*, lanes 1–3). In contrast, RT-PCR reactions using primers located in the 3′ end of the 25S sequence and just downstream from SP1 to detect read through transcription from an upstream gene did not produce any amplification band (data not shown).

In conclusion, these results suggest that accumulation of pre-rRNA in *Atnuc-L1* mutant plants is probably due to higher RNA pol I transcription from the IGS.

### AtNUC-L1 binds specific and transcriptionally active rRNA genes in the NORs

We previously reported that the AtNUC-L1 protein specifically binds rRNA gene promoter sequences and directs rRNA transcription from the TIS [18]. Accordingly, we would expect that AtNUC-L1 also binds the IGS and other rRNA coding sequences to activate or repress specific rRNA genes located in any of the NOR associated loci on chromosome 2 (*NOR2*) and 4 (*NOR4*) [35], [36].

**Figure 6:**
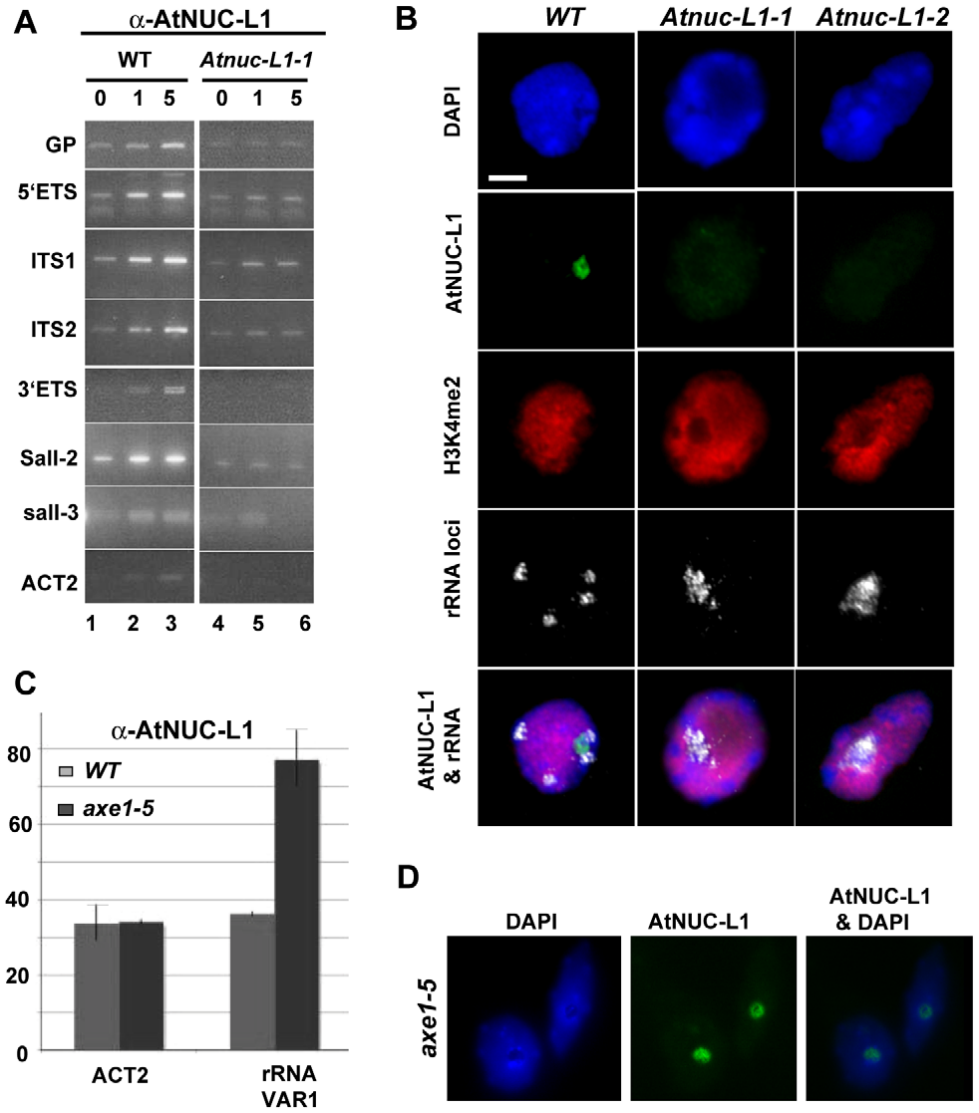
AtNUC-L1 binds specific rRNA gene units. A) Chromatin isolated from WT plants was incubated either with 0, 1 or 5 µl of α-AtNUC-L1 antibody -conjugated to Protein-A (lanes 1 to 3). Immunoprecipitated DNA was analyzed by PCR using specific primers to detect coding 5′ETS (*p22/*p23), ITS1 (*p24/p25*), ITS2 (*p26/p27*) and 3′ETS (*p9/p2*) and non-coding GP (*p20/p21*), Sal1-2 (*p28/p29*) and Sal1-3 (*p30/p31*) rRNA sequences. Lanes 4–6 correspond to PCR amplification using chromatin isolated from *Atnuc-L1-1* plants, which lack AtNUC-L1 protein, used as a control. Location of primers *p2*, *p9* and *p20-p31* on the rRNA gene is shown in Figure 1 and Figure 2. B) Co-localization of AtNUC-L1 and rRNA genes. Counterstaining with DAPI (blue); immunodetection of AtNUC-L1 (green); immunodetection of histone H3K4 dimethylation (H3K4^me2^; red), FISH with a 25S rRNA probe which reveals the 45S rRNA loci (white) and the merged image (AtNUC-L1 and rRNA) on nuclei from WT, *Atnuc-L1-1* and *Atnuc-L1-2* mutant plants. Bar  =  5 µm. C) Chromatin samples prepared from WT (grey bars) and *axe1.5* mutant (black bars) plants were immunoprecipitated with α-AtNUC-L1 antibody and amplified with specific primers (*p32/p33*) to rRNA gene *VAR1*. Amplification of the *ACT2* gene was performed to control ChIP and PCR reaction. D) Nucleolar localization of AtNUC-L1 in *axe5-1* mutant. Counterstaining with DAPI (blue); immunodetection of AtNUC-L1 (green) and the merged image (AtNUC-L1 & DAPI) is shown.

To investigate this hypothesis, we performed ChIP reactions with α-AtNUC-L1 antibody and purified chromatin samples from WT *A. thaliana* seedlings. As shown in Figure 6A, PCR reactions using increased amounts of ChIP fraction detect *GP* (*p20/p21*), *5′ETS* (*p22/p23*), *ITS1* (*p24/p25*), *ITS2* (*p26/p27*), *3′ETS* (*p9/p2*), *Sal1-2* (*p28/p29*) and *Sal1-3* (*p30/p31*) rRNA gene sequences. The amplification signals, GP, *5′ETS*, *ITS1*, *ITS2*, *3′ETS* and *Sal1-2* were proportionally dependent on the amount of α-AtNUC-L1 antibodies added to the ChIP fraction (lanes 1 to 3). Note that amplification of 3′ETS rRNA gene sequences reveals two distinct bands. In contrast, PCR reactions to detect *Sal1-3* rRNA sequences generated only a weak amplification signal that is not dependent on the amount of antibody added to the ChIP reaction. To control the specific ChIP reaction with α-AtNUC-L1, we performed PCR amplification reactions using ChIP fractions obtained from *Atnuc-L1-1* plants (lanes 4–6). As expected, in plants lacking the AtNUC-L1 protein, amplification signals are similar to background signals detected in WT plants (lane 1, without α-AtNUC-L1) and are not dependent on the amount of α-AtNUC-L1 antibodies added to the ChIP sample. Only a weak amplification band corresponding to actin sequences was amplified in WT ChIP samples (*ACT2* panel).

Considering that VAR1 represents ∼50% of the rRNA genes identified in the genome of *A. thaliana* (Figure 2A), we wanted to determine if AtNUC-L1 binds either all or some of the NOR in the nucleus of *A. thaliana* plants. We performed immunofluoresence and *in situ* hybridization experiments to answer this question. As shown in Figure 6B, AtNUC-L1 protein is detected only in the nucleolus of WT plants as a green hybridization signal (Panel AtNUC-L1). On the other hand, four rRNA signals are detected in interphase nuclei of WT plants (Panel rRNA). These signals probably correspond to the two *NOR2* and two *NOR4* expected in a diploid *thaliana* cell. In a preceding paper, we showed that absence of nucleolin in *Atnuc-L1* plants induces NOR decondensation [18]. Figure 6B of the present paper shows that in both *Atnuc-L1-1* and *Atnuc-L1-2* plants, the 45S rRNA chromatin is disorganized and invades the nucleolus. Superimposition of AtNUC-L1 and rRNA gene hybridization signals clearly shows that AtNUC-L1 co-localizes with only some (∼49%, see Table S1 for statistic analysis) of the rRNA loci (Panel AtNUC-L1 & rRNA). Additionally, AtNUC-L1 and rRNA gene signals co-localize only partially. Hybridization experiments using antibodies against H3K4^me2^, to examine global methylation status did not reveal major differences between WT and *Atnuc-L1* plant samples (panel H3K4^me2^). DAPI staining was visualized around the nucleolus (panel DAPI).

To determine whether or not AtNUC-L1 interacts with rRNA gene *VAR1* (inactive in WT plants), we performed ChIP using *A. thaliana* plants with a disrupted *HDA6* gene [14]. In *hda6* mutant plants (*axe1-5*) rRNA chromatin decondensation also induces expression of *VAR1* [16]. Subsequently, to study interaction of AtNUC-L1 with either active or inactive rRNA gene *VAR1*, we performed ChIP reactions using α-AtNUC-L1 antibodies and purified chromatin samples from WT and *axe1-*5 mutant plants. As shown in Figure 6C, qPCR reactions using primers *p32/p33* (shown in Figure S3) reveals specific interaction of AtNUC-L1 protein with *VAR1* in *axe-5* plants. Amplification of *ACT2* in WT and in *axe1-*5 plants shows that interaction of AtNUC-L1 with *VAR1* in WT is not specific. Indeed, amplification of *ACT2* sequences is similar to signals of *VAR1* rRNA gene in WT. We also demonstrated that expression of rRNA gene *VAR1* is not due to a delocalization or repression of AtNUC-L1 protein expression in the *axe1-5* mutant plants (Figure 6D). Inmunolocalization experiments show that AtNUC-L1 protein is detected in the nucleolus of *hda6* mutant plants as a green hybridization signal (Panel AtNUC-L1). DAPI staining was visualized around the AtNUC-L1 signal in the nucleolus (panel AtNUC-L1 & DAPI).

We conclude that AtNUC-L1 interacts with transcriptionally active rRNA genes. These experiments also corroborate the role of AtNUC-L1 in chromatin condensation and suggest that the AtNUC-L1 protein binds active rRNA genes located in two specific *NORs* (either *NOR2* or *NOR4*).

### Symmetric methylation, but not post-translational histone modifications patterns, are altered in *Atnuc-L1* mutants

In genetic hybrids, silencing of rRNA genes inherited from one parent requires DNA methylation and repressive histone modifications [37]. These modifications also involve specific small interfering RNAs (siRNAs) that direct specific DNA methylation [15]. In plants, DRM2 and CMT3 are responsible for methylation of CHH and CHG sites respectively, while MET1 maintains CG methylation [38].We tested if any of these features are involved in transcriptional activation and/or repression of the rRNA gene variants in *Atnuc-L1* mutant plants.

**Figure 7:**
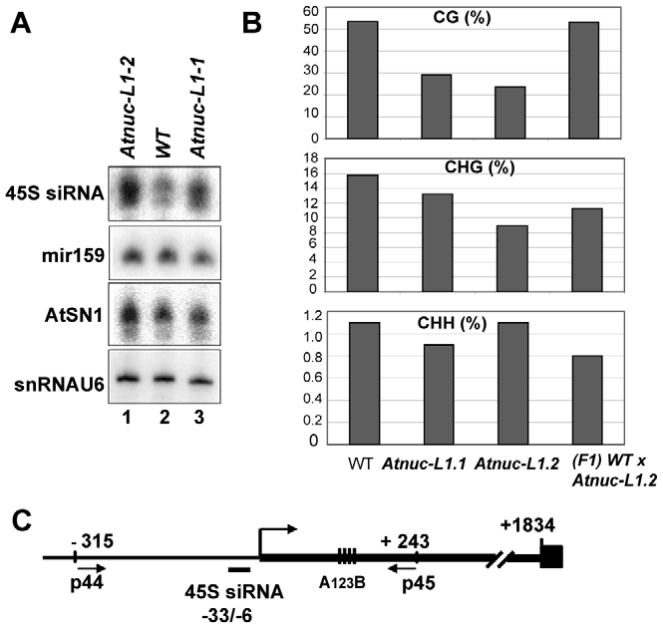
AtNUC-L1 gene disruption induces accumulation of siRNA 45S and rRNA gene hypomethylation on the 5′ETS. A) Total RNA from WT (lane 2) and Atnuc-L1 (lanes 1 and 3) plants were fractionated on PAGE and hybridized with [γ^32P^] 5′-end labeled primers to detect siRNA (45S, AtSN1) and miR159. Hybridization to detect snRNA U6 was performed to control RNA loading in each sample. B) Bisulfite sequencing analysis. The bar graphs show the representation (%) of methylated sites in the in 5′ETS rRNA gene sequences (from -+1 to +243) from WT, Atnuc-L1 mutant and F1 WT x Atnuc-L1-2 backcrossed plants in a CG (upper panel), CHG (middle panel) and CHH (lower panel) context. C) The schema shows the position of primers p44/p45 (located at −315 and at +243) and oligonucleotide 45S siRNA (from −33 to +6) used for sequencing bisulfite treated samples and for northern blot respectively. Conserved A^123^B motif in the 5′ETS is shown.

To determine if specific siRNAs to 45S rRNAs sequences accumulate in *Atnuc-L1* plants, we performed northern blot experiments using specific probes for promoter 45S rRNA sequences [39]. As shown in Figure 7A, the 45S siRNA probe hybridizes to a small RNA that accumulates ∼1.8 and ∼1.7 fold in *Atnuc-L1-1* and *Atnuc-L1-2* mutant plants respectively (lanes 3 and 1) compared with WT samples (lane 2). Higher levels of 45S siRNA were also observed in Northern blot experiments using total RNA extracted from WT and *Atnuc-L1* flower buds (data not shown). Hybridization with primers corresponding to *mir159* and *AtSN1* siRNA did not detect major differences between WT and *Atnuc-L1* samples (Panel miR159 and AtSN1). Hybridization with primers specific to small nuclear RNA U6 was used as an RNA loading control (panel snRNA U6).

To determine if siRNA accumulated in *Atnuc-L1* mutant plants affected rRNA gene methylation, we performed bisulfite sequencing assays of both rRNA gene promoter (Figure S11) and 5′ETS (Figure 7B) sequences. In the 5′ETS (from +1 to +243) we observed lower CG methylation levels (panel CG) in both *Atnuc-L1-1* (∼30%) and *Atnuc-L1-2* (∼24%) mutant lines compared with WT plants (∼54%). A significant decrease of CHG methylation level (∼16% in WT) was also observed in *Atnuc-L1-2* (∼9%) and to some extent in *Atnuc-L1-1* (13%) mutant lines (panel CHG). Remarkably, decreased CG and CHG methylation was mainly observed in the 5′ETS rRNA sequences but not in sequences located upstream from the TIS (from −315 to +1, Figure S11). We did not observe significant changes in the CHH methylation level in *Atnuc-L1* compared to WT plants (panel CHH), either in the promoter and/or in the 5′ETS regions (Figure 7B and Figure S11). Interestingly, we did not detect any major changes in the acetylation and methylation status of histone H3 in the rRNA genes from *Atnuc-L1* plants (Figure S6), nor did we observe significant differences in the methylation status in the immunodetection experiment shown in Figure 6B (panel H3K4^me2^). When the *Atnuc-L1-2* mutants were backcrossed with WT plants the CG methylation (53.2%) returned to WT levels (Figure 7B). In contrast, although the CHG methylation level increases (∼ 11.3%) in the F1 backcrossed plants, it remains lower compared with WT plants. Finally, in the F1 backcrossed plants the level of pre-rRNA return to those observed in WT plants (Figure S10).

We conclude that disruption of AtNUC-L1 affects accumulation of siRNA specific to 45S rRNA genes and symmetric methylation of 5′ETS rRNA sequences but does not affect the overall histone methylation status of 45S rRNA genes.

## Discussion

The present results provide evidence that disruption of *AtNUC-L1* gene expression affects RNA pol I transcription regulation at multiple levels. On one hand, by increasing RNA Pol I association with rRNA genes and, on the other, by switching the transcriptional state of specific rRNA gene variants.

The variations in the 45S rRNA genes detected in this work are in the 3′ETS sequences. We do not observe major variations in the promoter and/or in the 5′ETS rRNA gene sequences. Remarkably, among the four distinct 3′ETS rRNA gene variants present in the genome of *Arabidopsis thaliana*, the most highly represented (VAR1, ∼50%) is not expressed in WT but in *Atnuc-L1* mutant plants (Figure 2 and data not shown). In this context it would be interesting to determine if rRNA *VAR1* genes localize at NOR2 or NOR4 or at specific heterochromatic positions in the NORs.

We also observed that the rRNA variants are differentially expressed in *A. thaliana* WT plants. However, activation and/or repression of these rRNA variants are probably more dependent on the developmental stage and/or tissue rather than cell growth conditions (Figure 3). Remarkably, we detected rRNA *VAR1* gene expression essentially in germinating seeds and *Atnucl-L1* mutants dependent, i.e. when chromatin is decondensed (Figure 3B and Figure 6B). Developmental activation and repression of rRNA genes is also observed in genetic hybrids. For instance, in allotetraploid *B. napus*, the *B. oleracea*-derived rRNA is normally repressed and *B. rapa*-derived rRNA is active in vegetative tissue. However, rRNA transcripts from *oleracea* are found in all organs derived from floral meristems, suggesting that the *oleracea* genes silenced during vegetative growth are derepressed on flowering [40]. Similarly, developmental modulation of dominant and underdominant rRNA genes was also observed in *Solanum* allopolyploid species [41] and *Arabidopsis* hybrids [29].

Higher level of RNA pol I transcription of rRNA genes in the absence of AtNUC-L1 is supported by an increased amount of RNA pol I subunit associated with rRNA genes in *Atnuc-L1* plants (Figure 5A). In agreement, *in situ* hybridization experiments confirm that accumulation of pre-rRNA is correlated with a higher concentration of RNA polymerase subunit in the nucleolus of *Atnuc-L1* plants (Figure S5). Thus, it is possible that accumulation of pre-rRNA is due to a higher loading of RNA pol I subunits and/or an increased number of active rRNA genes. Interestingly, the level of preRNA transcripts from GP decreases in the *Atnuc-L1* mutant (Figure 5B). Thus, accumulated pre-rRNAs are probably RNA pol I products transcribed from the IGS rather than the TIS in the rRNA gene promoter (Figure 5C). In agreement with these results, a previous report showed transcription from spacer promoter rRNA sequences in *Arabidopsis thaliana* [9]. Although the accumulation of pre-rRNA transcripts could also be generated by deficiency of the pre-rRNA machinery in *Atnuc-L1* plants, our results indicate that this is not case. Indeed, fibrillarin remains localized in the disorganized nucleolus of *Atnuc-L1* plants and pre-rRNA processing events are still accurate (Figure 4, Figure S4 and S5). However we do not discard the possibility that the rate of pre-rRNA processing activity of standard or longer pre-rRNA could be affected en absence of nucleolin.

RNA Pol I transcription from the IGS was also observed in mouse cell culture [42]. In this case, transcription from the IGS is dependent on the amount of TIP5, the large subunit of the chromatin remodeling complex NoRC [43]. As in *Atnuc-L1*, depletion of TIP5 decreases the level of promoter -initiated transcripts and leads to accumulation of pre-rRNA. However, in contrast to TIP5, disruption of AtNUC-L1 does not affect heterochromatic marks (Figure 6 and Figure S6). This suggests that AtNUC-L1 determines assembly or positioning of nucleosomes rather than chromatin epigenetic changes. Indeed, mammalian nucleolin binds histone H1 [44], [45] and play a role in histone chaperoning and assisting remodeling of nucleosomes [23]. A role in chromatin remodeling has also been demonstrated for nucleolar transcription factor UBF (Upstream Binding Factor) in animals. The UBF factor displaces histone H1 from nucleosomes [46], controls rRNA transcription [47] and was recently reported to determine the number of active genes by a methylation-independent mechanism [34]. Despite these similarities, AtNUC-L1 and UBF are structurally different proteins and consequently they might affect rRNA gene activation/repression through different molecular mechanism.

AtNUC-L1 co-localizes with only approximately half of the rRNA loci detected in the nucleus of WT plants (Figure 6 and Table S1). In *Arabidopsis thaliana* plants, rRNA genes are located at two *NORs*, one on chromosome 2 and the other one on chromosome 4 [35], [36]. We can not say if AtNUC-L1 binds NOR2 and/or NOR4. However, we predict that AtNUC-L1 only associates with potentially transcriptionally active rRNA genes located at one of the two NORs. This is based on the fact that nucleolin localizes in the nucleolus, which is a consequence of rRNA gene expression [1], [3], [48]. Moreover, while almost all the AtNUC-L1 signal is in the nucleolus, most of the rRNA signal is located in its periphery in the knob structures of condensed and inactive rRNA chromatin [49]. In addition, ChIP and inmunolocalization experiments using a plant mutant (*axe1-5*) in which the rRNA gene *VAR1* is active (recently reported in [16]), also indicate that AtNUC-L1 binds transcriptionally active rRNA genes (Figure 6). Interaction of AtNUC-L1 with active rRNA genes is also supported by ChIP experiments that demonstrate that AtNUC-L1 co immunoprecipitates mainly with active *A. arenosa* –derived rRNA genes rather than repressed *A*. *thaliana* –derived rRNA genes in *Arabidopsis suecica* hybrid plants (Figure S9).

Although there is accumulation of 45S siRNA in the *Atnuc-L1* plants (Figure 7), we did not observe major quantitative changes in the methylation and acetylation status of histone H3 in *Atnuc-L1* mutant plants that might indicate higher or lower number of active or repressed rRNA genes in *Atnuc-L1* mutant plants (Figure S6). In contrast we observed lower CpG methylation specifically in the 5′ETS region of the rRNA gene (Figure 7B) but not in the promoter region (Fig S11). Previous analyses of pericentromeric repeats and rRNA genes in *Arabidopsis* revealed that DNA methylation loss at CG dinucleotides persists into successive sexual generations [50]–[52]. Even when recessive mutations in genes required for CG methylation maintenance (e.g., DDM1 or MET1) are replaced by functional alleles, methylation within most repeats and transposons does not recover, or “reset” [53]. In contrast, our experiments with *Atnuc-L1-2* documented a full recovery of CG methylation in rRNA genes after backcrossing this mutation with wild type Col0 (Figure 7B). We hypothesize that AtNUC-L1 deficiency impairs CG methylation maintenance without abolishing the underlying chromatin state required for DNA methylation to be reestablished. On the other hand, only a partial recovery of CHG methylation was observed in F1 backcrossed plants. A similar situation was also observed in *hda6* mutants. Here, lost of CHG (and CG) methylation in the rRNA promoter region was also only partially recovered in the mutant plants rescued by the HDA6 transgene [16]. But in *Atnuc-L1* mutant plants, the complete recovery of CG methylation is sufficient to restore wild-type expression of intergenic transcripts and rRNA gene variant expression (Figure S10). Interestingly, chromatin immunoprecipitation using antibodies that recognize dimethylated H3K9 reveal no significant change between wild-type Col-0 and *Atnuc-L*1 mutants (Figure S6). Taken together, H3K9 dimethylation is potentially the underlying chromatin state that allows CG methylation to be reestablished efficiently in *Atnuc-L1* mutant heterozygote plants. This is not the case for pericentromeric repeats or rRNA genes after *ddm1* or *met1* back-crossing, but these mutants do show a loss of H3K9 dimethylation.

**Figure 8:**
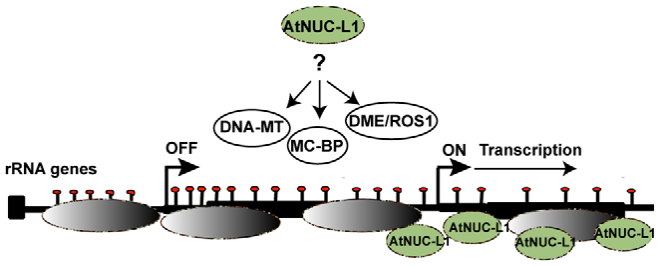
AtNUC-L1 controls symmetrical DNA methylation and nucleosome positioning to determine the “on” or “off” state of transcriptionally active rRNA genes. In WT plants AtNUC-L1 (in green) binds chromatin and 5′ETS rRNA sequences. AtNUC-L1 binding to the 5′ETS might maintain a suitable gene methylation pattern (red bullets) to set nucleosome (in grey) positioning and transcription from the GP. In this model, AtNUC-L1 might be required for nucleolar localization, assembly and/or activity of DNA-MT, MC-BP or DME/ROS1 protein complexes that repress or active rRNA transcription of specific loci in WT plants.

A few years ago we reported a plant nucleolin containing -U3snoRNP complex that specifically binds four conserved motifs in the 5′ETS (A^123^B, shown in Figure S8) and proposed a role for this complex in coupling transcription and processing of pre-rRNA [54], [55]. Here we demonstrate that AtNUC-L1 interacts directly with 5′ETS rRNA gene sequences but does not co-precipitate with RNA Pol I subunits (Figure S8). Thus, considering the nucleosome remodeling activity of nucleolin proteins [23], [56], it is rational to propose that binding of AtNUC-L1 to rRNA genes may be required to position nucleosomes in specific transcriptional frames that determine the ‘on’ or ‘off’ state of transcriptional active rRNA genes (Figure 8). Accordingly, demethylation of the 5′ETS rRNA gene sequences and activation of VAR1 suggest that AtNUC-L1 maintains gene methylation required for accurate nucleosome position for transcription (Figure 8). Indeed, several studies point to a major role of CpG methylation in nucleosome positioning and binding of multiples protein factors could be involve in repositioning of nucleosome [57], [58]. In this context, AtNUC-L1 activity might be required for rRNA gene methylation or demethylation to repress or activates RNA Pol I transcription. We do not know if AtNUC-L1 interacts with any DNA Methyl transferase (DNA-MT), demethylases (DME/ROS1) and/or Methyl cytocine –Binding protein (Mc-BP) (For references [59], [60]). However, nucleolin like proteins in plants interact in a large Ribo Nucleoprotein Complex (RNP) with the RNA methyltranferase fibrillarin (Figure S8) and other DNA/RNA modification activities, including RdRP and TSN proteins, which have been implicated in chromatin silencing [55].

Many questions remain, including mutual dependency and complex crosstalk among nucleolin-like proteins and other functionally related protein factors, such as HDA6 which has also been recently implicated in reactivation of rRNA gene VAR1 [16]. Finally, the AtNUC-L2 protein induced in *Atnuc-L1* mutant plants ([18] and Figure S1) might also play a major role in controlling activation and/or repression of these rRNA gene variants. However, in WT plants we have not been able to find any correlation between AtNUC-L2 and rRNA *VAR1* gene expression and consequently the physiological impact of a second nucleolin protein gene in plants remains an open question.

## Materials and Methods

### Plant growth conditions and mutant isolation

Seeds corresponding to *Atnuc-L1-1* and *Atnuc-L1-2* plants lines (SALK_053590 and SALK_002764 respectively) were obtained from the Nottingham *Arabidopsis* Stock Center (http://nasc.life.nott.ac.uk). *Atnuc-L1-1* was reported previously in [18] and *Atnuc-L1-2* corresponds to the *parl1-2* allele described by [28]. The plant mutant *axe1-5* was provided by C. Pikaard and described in [16]. To obtain backcrosses *WT x Atnuc-L1-2* plants, pollen from *Atnuc-L1-2* was used to fertilize WT Col0 plants. Then F1 heterozygous plants where selected by PCR as described previously in [18].

### RNA extraction and blot analysis

Total RNA was isolated using TriZol reagent (GE Healthcare, Littler Chalfont, Bukimhamshire, UK) as described previously [18]. Total RNA (15 µg) was either fractionated on 0.8% formaldehyde agarose gels and transferred to nitrocellulose (Hybond+, GE Healthcare) or fractionated on 15% polyacrylamide gels and transferred to Hybond NX (GE Healthcare). For detection of pre-rRNA precursors and small RNA, 10 pmoles of oligonucleotide primers were 5′end labeled using 50 µCi of [γ^32^ P] ATP (6000 Ci/mmol) and T4 PNK (Promega). Oligonucleotides probes are listed in the supplementary material (Text S1). After overnight hybridization at 42°C (in 50% formamide, 0.5% SDS, 6X SSC, 5% Denhardt's solution buffer), filters were washed once 15 min. at 42°C with 2X SSC, 0.1% SDS and twice 15 min. at 65°C with 2X SSC, 0.1% SDS before exposure (over night for *p34*, p35, *p37*, *p39* and *p41* and 3 hrs for p36, *p3*8 and *p40*) in a PhosphoImager (Molecular Dynamics).

### Amplification and cloning of 3′ETS rRNA and pre–rRNA sequences

For cloning 3′ETS rRNA sequences, genomic DNA extracted from WT and *Atnuc-L1* plants was amplified using GoTaq DNA polymerase (Promega). For cloning 3′ETS pre-rRNA, the RT-PCR reaction was performed using total DNA free- RNA (∼1 µg) and a OneStep RT_PCR Kit (QIAGEN). Corresponding pairs of specific primers used for amplification are indicated in each figure. The amplified rRNA and pre-rRNA sequences were then cloned in a pGemT easy vector (Promega). All clones were sequenced with a model 3130 DNA sequencer and an ABI PRISM Big Dye Terminator Cycle Sequencing Ready Reaction Kit (Applied Biosystems, Foster City, CA).

### Primer extension and RNase A/T1 protection assay

Primer extension analysis was performed using total RNA (15 µg) and specific 5′-end labeled primers as previously described [18]. Primers used were: *p3* for detection of primary pre-rRNA precursor (+104 nucleotides from TIS) and *3AtU3* for detection of U3snoRNA used as loading control. Dideoxy sequencing reactions were performed using the *fmol* DNA Cycle Sequencing System (Promega) with a pGem-3Z plasmid vector containing the *A. thaliana* rRNA sequences from −520 to +1940 [61].

For RNase A/T1 mapping, the 3′ETS rRNA sequence that includes 16 nucleotides of the 3′ end of 25S rRNA followed by 664 bases of non coding sequence, was amplified from genomic DNA using primers *p1* and *p4* and cloned in the pGemT easy vector (Promega). The riboprobe was produced by *in vitro* transcription of the linearized antisense rRNA sequence by incorporating [α^32^P] CTP. The radiolabeled RNA probe was purified on 8% PAGE and the RNase protection assay performed as described previously [54].

### Immunostaining and FISH

For the combination of immunostaining and FISH, immunodetection was performed prior to FISH. Young rosette leaves were squashed on slides in PBS 1X / 4% paraformaldehyde. Immunodetection of AtNUC-L1 was performed using α-AtNUC1 primary antibody followed by detection with the fluorochrome Alexa 488 - conjugated goat anti-rabbit IgG (Sigma). H3K4me2 immunodetection was performed using an anti-H3K4me2 primary antibody (Upstate) followed by detection with the fluorochrome Alexa 594 - conjugated goat anti-rabbit IgG (Sigma-Aldrich, St. Louis, MO). FISH was subsequently performed as described previously [18]. Nuclei were stained with DAPI (4′, 6-diamidino-2-phenylindole) mounting medium (Vectashield, Vector Laboratories). A more detailed description of this immuno-FISH experiment is available in the supplementary information section (Text S1). Immunodetection of fibrillarin was performed using mouse fibrillarin (72B9) primary antibody prior to detection with the fluorochrome Alexa 488 - conjugated goat anti-rabbit IgG (Sigma). For image acquisition, an epifluorescence Imager Z1 microscope (Zeiss) with an Axiocam MRm camera (Zeiss) was used.

### Chromatin immunoprecipitation

Chromatin immunoprecipitation (ChIP) was performed as described previously [18], [62]. To immunoprecipite rRNA chromatin associated with AtNUC-L1 protein, rabbit polyclonal antibodies against the C-terminal peptide sequence of AtNUC-L1 (Figure 4C) were custom made by NeoMPS (Strasbourg, France).

### Real-time quantitative PCR

DNA was amplified using an Applied Biosystems model 7500 thermocycler with 0.5 units of Platinum Taq (Invitrogen), SYBR Green I (Invitrogen), and Internal Reference Dye (Sigma). Results were analyzed using the comparative C_T_ method (Livak and Schmittgen, 2001) relative to input.

### Bisulfite analysis

Genomic DNA from WT, *Atnuc-L1* mutant and F1 *Atnuc-L1-2 x W*T plants was extracted using Illustra DNA extraction kit phytopure (GE Healthcare) following the manufacturer's instruction. 2 µg of DNA was digested overnight using 20 Units of BamHI restriction enzyme prior to bisulfite conversion. Bisulfite treatment was performed using Epitect Bisulfite kit (Qiagen). The primers *p42* and *p43* were used to amplify rRNA gene sequences (from −315 to +243) and resulting PCR products were clone in the pGemT easy vector (Qiagen). Between 31 and 41 clones per sample (WT, 41; *Atnuc-L1-1*, 35; *Atnuc-L1-2*, 31) were sequenced and analyzed using the CyMATE method [63].

## Supporting Information

Figure S1 Similar growth and molecular phenotypes of two independent *Atnuc-L1* mutant plants. A) Top, picture of WT, *Atnuc-L1-1* and *Atnuc-L1-2* plants grown on soil ∼4 weeks under 16:8 –h (light: dark) cycle. The bar graph shows shoot fresh weight (in grams per plant) from WT and *Atnuc-L1-1* and *Atnuc-L1-2* plants. Bottom, diagram of the *AtNUC-L1* gene from the ATG start to the TGA stop codon. The black boxes correspond to exons separated by 14 introns. The T-DNA insertion in the *Atnuc-L1-1* (salk_053590) and *Atnuc-L1-2* (salk_002764) plants is indicated. B) Top, PCR reactions using cDNA prepared from RNA isolated from WT (lane 1) and *Atnuc-L1* (lanes 2 and 3) plants to detect *AtNUC-L1* transcripts. *eIF1α* gene expression was analyzed to evaluate the amount of cDNA used in each reaction. Bottom, the level of *AtNUC-L2* transcripts in both *Atnuc-L1* mutant plants was determined by qPCR. C) Western blot analysis using specific antibodies against the AtNUC-L1 and AtNUC-L2 peptides and total protein extracts from WT and *Atnuc-L1* mutant plants. The diagram shows the sequence and position of the peptide in the C-terminal region of protein AtNUC-L1. See [17] for PCR and western blot conditions.
(0.71 MB TIF)

Figure S2 Complementation studies. A) Top, plant growth and development defects in mutant plants are fully restored in transformed *Atnuc-L1* mutant plants: a, WT; b and c, *Atnuc-L1-1 Atnuc-L1-2*, d and e transformed *Atnuc-L1-1 Atnuc-L1-2* plants. Bottom, *Atnuc-L1* mutant plants were transformed with *AtNUC-L1* genomic sequences fused to epitope tag flag-HA. Genomic DNA includes 1.1 kb sequence upstream from staring ATG and AtNUC-L1 coding region (exons and introns minus TGA stop codon).B) RT PCR reaction to detect rRNA variants (3′ETS rRNA panel) in WT (lane 1), *Atnuc-L1* (lanes 2 and 3) and complemented plants (lanes 4 and 5). Amplification of *eIF1α* mRNA was used to controls total amount of RNA in each sample (Panel eIF1α). Amplification of eIF1α genomic DNA shows absence of DNA contamination in the RNA samples (lane 6). C) Western blot analysis using specific antibodies against the AtNUC-L1 peptide and total protein extract from WT (lane 1), *Atnuc-L1* (lanes 2 and 3) and transformed plants (lane 4 and 5). The same membrane was hybridized with antibodies against a NADPH Thioredoxine Reductase (NTR) to verify similar amount of protein in each sample.
(0.31 MB TIF)

Figure S3 Alignment of 3′ETS rDNA sequences cloned either by PCR using genomic DNA (VAR1, VAR 2 and VAR3) or by RT-PCR using total RNA (VAR4) from WT plants. The grey solid line shows the 3′end of the 25S rRNA sequence and the black solid lines repeat (R1-R4) sequences located downstream of 3′ETS cleavage site detected by RNase mapping. The double line just upstream of R2, shows the position of the triplet CAC missed in the VAR2a and VAR3a isoforms (not shown). Primers used to detect specific rRNA variants (*p32/p33* and *p1/p5* for VAR1 and VAR4 respectively) and all rRNA variants (*p3/p4*) are shown by dotted arrows.
(0.97 MB TIF)

Figure S4 Processing of accumulated pre-rRNA in *Atnuc-L1* mutant plants is accurate. A) Northern blot analysis using total RNA isolated from WT and *Atnuc-L1* mutant plants and [γ^32^P] 5′-end labeled primers (*p34-p41*) to detect 5′ETS1 (*p34*, lanes 1–3), 5′ETS2 (*p35*, lanes 4–6), 18S (*p36*, lanes 7–9), ITS1 (*p37*, lanes 10–12), 5.8S (*p38*, lanes 13–15), ITS2 (*p39*, lanes 16–18), 25S (*p40*, lanes 19–21) and 3′ETS (*p41*, lanes 22–24) pre-rRNA sequences. B) The agarose gel stained with GelRed (BioTium) show similar amounts of total RNA in WT (lane 3) and *Atnuc-L1* (lanes 1 and 2) RNA samples. C) The diagram represents a model of pre-rRNA processing steps and rRNA mature and intermediates detected both in WT and *Atnuc-L1* mutant plants. The smear detected with *p34* might correspond to an exonucleolytic trimming of the 5′end product from 5′ETS cleavage in the P site, while the signal indicated by asterisk the an rRNA intermediates generated by an alternative cleavage upstream of P site (depicted by a vertical arrow). Panels 5ETS1, 5ETS2 18S and 3ETS (shown in figure 4) are presented here to pinpoint position of intermediated and mature rRNA.
(0.75 MB TIF)

Figure S5 Accumulation of RNA Pol I sub unit and pre-rRNA in *Atnuc-L1* mutant plants. Left, *in situ* hybridization of pre-rRNA in nucleoli of roots from WT and *Atnuc-L1-1*. Hybridization was performed using a pre- rRNA probe (shown in the Fig.) to detect 5′ETS uncleaved pre-rRNA. Right, Inmunolocalization of RNA polI subunit in WT and *Atnuc-L1-1* plants using antibodies against the 24.3 kDa subunit. Bar = 10 µm. In Panel pre-rRNA, the accumulated small punctuate loci dispersed throughout the nucleolus of *Atnuc-L1* plants can be compared with the multiple transcriptional initiation sites detected by BrU incorporation in pea and other species [6]. In Panel α-24.3, we observed a localization of the 24.3 kDa subunit in “focus” both in the nucleolus of WT and *AtnucL1* plant mutants (Panel α-PolI). Although in the nucleolus of *Atnucl-L1* plants these “foci” are less numerous they are larger in size compared with those observed in the nucleolus of WT plants.
(0.49 MB TIF)

Figure S6 AtNUC-L1 gene disruption does not affect histone epigenetic marks. A. Chromatin samples prepared from WT (light grey bars), *Atnuc-L1-1* (dark grey bars) and *Atnuc-L1-2* (black bars) plants were inmunoprecipitated with antibodies against H3Ac, H3K4^me2^ and H3K9^me2^. ChIP samples were then amplified with specific primers to 5′ETS (p12/p13), 25S (p14/p15), 3′ETS (p16/p17) and IGS2 (p18/p19) rRNA gene sequences. Amplification of ACT2 and soloLTR gene was performed to control ChiP and qPCR reactions.
(0.35 MB TIF)

Figure S7 rRNA VAR1 gene expression in *Atnuc-L1-1* mutant plants. RT PCR reaction was performed with total RNA prepared from individual WT (lanes 1–5) and *Atnuc-L1-1* (lanes 6–10) mutant plants and using primers *p3-p4* to detect 3′ETS rRNA variant expression. Amplification of *eIF1α* mRNA was used to controls total amount of RNA in each sample (Panel eIF1α). Amplification of eIF1α genomic DNA shows absence of DNA contamination in the RNA samples (lane 11).
(0.12 MB TIF)

Figure S8 AtNUC-L1 binds 5′ETS but it does not interacts with RNA Pol I. A) Analysis of 5′ETS binding activity on 4% polyacrylamide gel. EMSA was performed with 1, 5, 10 and 15 µl (lanes 2–5) of recombinant His-AtNUC-L1 protein (0.6 µg/ml) protein and with a α-^32^P-dCTP filling Klenow labeled rDNA probe. Lane 1, rDNA probe alone B) Inmunoprecipitation (IP) was performed with total protein extracts and antibodies against AtNUC-L1 coupled to agarose beads. After IP reaction total (lane1), unbound (lane 2) and bound (lane 3) proteins were subjected to PAGE and analyzed by Western blot using antibodies against AtNUC-L1, the 24.3 kDa subunit of RNA Pol I and fibrillarin from *Arabidopsis* (AtFib). C) Diagram of the rDNA probe A^123^B used in the EMSA assay. The probe encompasses genomic 5′ETS rDNA nucleotides from +96 to +327.
(0.12 MB TIF)

Figure S9 AtNUC-L1 binds actives rRNA genes. Chromatin samples prepared from the allotetraploid *Arabidopsis suecica* (where the *thaliana* derived rRNA genes are repressed and those of *arenosa* are expressed) were inmunoprecipitated with antibodies against AtNUC-L1. The 5'ETS sequences were detected by PCR using primers *p46/p47* and labeled primers *p48* or *p49* specific to 5′ETS sequences from *A. thaliana* or *A. arenose* respectively. Amplification of *ACT2* gene was performed to control ChiP and qPCR reactions. The qPCR reactions were performed using the TaqMan system (Premier Biosoft).
(0.03 MB TIF)

Figure S10 pre-rRNA transcripts levels in backcrossed F1 WT x *Atnuc-L1-2* plants. RT PCR reaction to detect pre- rRNA transcripts in WT (lane 1), *Atnuc-L1-2* (lanes 2) and *F1 WT x Atnuc-L1-2* (lane 3) plants Amplification was performed with primers *p3/p4* to detect 3′ETS rRNA variants (Panel 3′ETS VAR), primers *p7/p8* and *p42/p43* to detect transcripts from IGS (Panels −223/+243 and −507/−201 respectively). Amplification of actin mRNA (Panel *ACT2*) was used to control total amounts of RNA in each sample. No amplification bands were detected in RT-PCR reactions without reverse transcriptase
(0.27 MB TIF)

Figure S11 Bisulfite sequencing analysis. The bar graphs show the representation (%) of methylated sites in the rRNA gene promoter sequences (from −315 to +1) from WT and *Atnuc-L1* mutant plants in a CG (upper panel), CHG (middle panel) and CHH (lower panel) context. The schema with the position of primers *p44*/*p45* (located at −315 and at +243) used for sequencing bisulfite treated samples is shown in figure 7.
(0.08 MB TIF)

Table S1 Statistical analysis of AtNUC-L1 protein and FISH signal detected either in WT and/or *Atnuc-L1* mutant plants.
(0.03 MB DOC)

Text S1 Supporting text.
(0.07 MB RTF)
