# Supplementary material for: Nucleolin Is Required for DNA Methylation State and the Expression of rRNA Gene Variants in Arabidopsis thaliana
Source: PLoS Genet. 2010 Nov 24;6(11):e1001225. doi: 10.1371/journal.pgen.1001225 (PMC2991258; doi:10.1371/journal.pgen.1001225)
Supplement: Table S1 — Statistical analysis of AtNUC-L1 protein and FISH signal detected either in WT and/or Atnuc-L1 mutant plants. (0.03 MB DOC) [file pgen.1001225.s012.doc]

## Table S1

| Cytological analysis of 45S rDNA and AtNUC-L1 in WT nuclei | |
| --- | --- |
| Mean number of AtNUC-L1 signals per nucleus  Standard deviation | 1.1  ±0.044 |
| Mean number of rDNA signals per nucleus  Standard deviation | 2.3  ±0.105 |
| Proportion of rDNA signals associated with AtNUC-L1  Standard deviation | 48.9%  ±0.074 |
| Number of nuclei analyzed | 48 |

**Table S1.** Statistical analysis of AtNUC-L1 protein and FISH signal detected either in WT and/or *Atnuc-L1* mutant plants.
