# Supplementary material for: Nucleolin Is Required for DNA Methylation State and the Expression of rRNA Gene Variants in Arabidopsis thaliana
Source: PLoS Genet. 2010 Nov 24;6(11):e1001225. doi: 10.1371/journal.pgen.1001225 (PMC2991258; doi:10.1371/journal.pgen.1001225)
Supplement: Text S1 — Supporting text. (0.07 MB RTF) [file pgen.1001225.s013.rtf]

TEXT S1
Plant material 
All lines were derived from Arabidopsis thaliana Columbia (Col 0) ecotype. Seeds were first sowed on Murashige and Skoog medium (MS) or soil and left 2 days at 4°C to synchronize. Plants were then grown either under continuous light (MS plants) or under a 16h light/ 8h dark cycle (soil plants).
Northern blot conditions
For detection of 5'ETS and 3'ETS pre-rRNA precursors, RNA blot hybridizations were carried out overnight at 50°C in 50% formamide, 0.5% SDS, 6X SSC, 200mg/mL DNA salmon sperm DNA, 5X Denhart's solution and p1 and p2 end-labeled primers. 
For detection of small bio-regulator RNAs, pre-hybridization and hybridization was carried out in 5× SSC, 20 mM Na2HPO4 pH 7.2, 7% SDS, 2× Denhardt solution, 50 mg/ml denatured herring DNA at 50°C. Filters were washed twice with 3X SSC, 25 mM NaH2PO4 pH 7.5, 5% SDS at 50°C for 10 min, followed by one to two washes with 1× SSC, 1% SDS at 50°C.
Immunostaining and FISH 
For the combination of immunostaining and FISH, immunodetection was performed prior to FISH. For image acquisition, an epifluorescence Imager Z1 microscope (Zeiss) with an Axiocam MRm camera (Zeiss) was used. Fluorescence images for each fluorochrome were captured separately through the appropriate excitation filters. Young rosette leaves were squashed on slides in PBS 1X / 4% paraformaldehyde and slides were washed twice in 1X PBS. Slides were blocked in 2% BSA in 1X PBS (30 min, 37°C), incubated with -NUC1 primary antibody (dilution 1:500 in 1% BSA/1X PBS) overnight at 4°C. Slides were washed in 1X PBS and detection performed with the fluorochrome Alexa 488 - conjugated goat anti-rabbit IgG (Sigma). After 1X PBS washes, nuclei were stained with DAPI (4', 6-diamidino-2-phenylindole) mounting medium (Vectashield, Vector Laboratories). Images of nuclei were captured and positions recorded. For the second immunodetection (H3K4me2), DAPI medium was first removed by 1X PBS washes, then slides were washed in glycine buffer pH2 (30 min) and in 1X PBS. Prior to incubation with the primary -H3K4me2 antibody (1:250 in 1% BSA/1X PBS, 1h at 37°C) (Upstate), slides were blocked in 2% BSA in PBS 1X (30 min, 37°C). They were washed in 1X PBS and detection performed with the fluorochrome Alexa 594 - conjugated goat anti-rabbit IgG (Sigma-Aldrich, St. Louis, MO). After 1X PBS washes, nuclei were stained with DAPI (4', 6-diamidino-2-phenylindole) mounting medium (Vectashield, Vector Laboratories) and images recorded. The slides were washed in 1X PBS to remove DAPI mounting medium. After postfixation in 2% paraformaldehyde in 1X PBS, slides were air-dried. Subsequently, they were baked at 60°C, treated with RNase (100 µg/mL in 2x SSC) at 37°C for 1 h, then with pepsin (10 µg/mL in water, pH 2) at 37°C for 20 min, washed again in 1X PBS, dehydrated in an ethanol series and air-dried. Hybridization, washing and detection of the labeled probe were performed as for FISH on spread nuclei as described previously (Pontvianne et al. 2007). Images were captured, pseudocolored, merged and processed with the Adobe Photoshop software (Adobe Systems). 48 to 55 nuclei were analyzed for each genotype. Inmunodetection of fibrillarin was performed using antibodies against mouse fibrillarin (72B9) and secondary antibody conjugated to Alexa-488. 
Chromatin Inmunoprecipitation.
Chromatin immunoprecipitation (ChIP) was performed as described previously (Pontvianne et al. 2007; Wierzbicki et al. 2008). Antibodies against H3K9me2 (ab7312), H3K4me2 (ab11946) and H3 acetyl K9 (ab4441) were obtained from Abcam. The GP (Gene Promoter), 5ETS (5'External Transcribed Spacer); ITS1 (Internal Transcribed Spacer 1), ITS2 (Internal Transcribed Spacer 2); 3ETS (3'External Transcribed Spacer), 25S (25S rRNA), IGS2 (Inter Genic Spacer2), Sal1-1 and Sal1-3 (Sal repeat elements 1 and 3) sequences were amplified using specific primers p10–p33 as indicated in Fig.1. 

Primers used in this work  (Lab's name): 

p1. GTCGCTAAGATTCGAC (25S3'ETS(2))
p2. CCTCGGACCCGGTAAAC (3At3ETS)
p3. GACAGACTTGTCCAAAACGCCCACC (5allrRNAvar)
p4. CTGGTCGAGGAATCCTGGACGATT (3allrRNAvar)
p5. CTCTTTTGTTTCTTTTTGAACAAGTC (3'rRNA4a)
p6. CCTCGTCCATCCC (At104)
p7. caactagaccatgaaaatcc (Alu5')
p8. caagcaagcccattctcctc (Alu3')
p9. CCCCAATTCTACACAAG (5At3ETS2)
p10: TTTACCAGAAAATAGGATTTAGTATCCTTATG (TIS-For)
p11: CGGTGCCAAGAAAAAAGAAATACTT (TIS-Rev)
p12: TTGGAACGATTGATGATTTTGAGT (p5'ETS-For)
p13: GCCTACGAACACTAGCTATCCGATC (5'ETS-Rev)
p14: TGTTCACCCACCAATAGGGAA (25S-For)
p15: TCAGTAGGGTAAAACTAACCTGTCTCAC (25S-Rev)
p16: GAATTCCCAACTTTACACGAGCTC (IGS1-For)
p17: AAGTAATAACATTTAACCTCGAGAGACGAG (IGS1-Rev)
p18: GTCAAACACTTGGTGATATGAACACA (IGS2-For)
p19: GCATGGGTTTGTCATATTGAACGT (IGS2-Rev)
p20: GTATCCTTATGATGCATGC (5AtProm110)
p21: CCTATATAGCTTAATAGCC (3AtProm3)
p22: GGCACGAGGAATGACCGACCG (5a123b)
p23: GAGAACTGCTGAGAAAACTCGG (3a123bb)
p24: GAACGACCCGCGAAC (5FITS1)
p25: GACTTCAGTTCGCAGCAC (3RITS1)
p26: TCGTCGTCCCTCACCATC (5FITS2)
p27: CCGTTACTAAGGGAATCC (5R25S)
p28. ctccatagagttgttttctc (5'Sal2)
p29. CTCTTGGCACCAACATAC (3'Sal2)
p30. CTCAATCGGGTCCGAGG (5'Sal3)
p31.  CATTGCATCATATCCAATG (3'Sal3)
p32.  ctcgaggttaaatgttattacttggtaagattccgg (5rRNA-VAR1)
p33. TATGCACCTTCGTGAGCGTC (3rRNA VAR1)
p34: GCCGATATCCGATACCATCCCTCGATCGCTA (At5ETS1)
p35: GCATTCATCGATCACGGCAATTCCCCGCCAC (At5ETS2)
p36: GTCCCTTCCATAAGTCGGGGTTTGTTGCACG (At18S)
p37: AGACTTCAGTTCGCAGCACAGCATCCGCCCA (AtITS1)
p38: ATTTCGCTACGTTCTTCATCGATGCGAGAGC (At5.8)
p39: CACCACCGCATGTCGGTACGCTCCAGGCGTC (AtITS2)
p40: CCGATTTTCAAGCTGGGCTCTTCCCGGTTCG (At25Sa)
p41 GGGGATTGTTTTTTTTGGAGTGATTTAGGGG (At3ETSA)
p42 ctaggtcatttgacctgatacaacatc (3TIS-201)
p43 aggacggcggaaccctcga (5TIS-507
p44: GGATATGATGYAATGTTTTGTGATYG (BS AmpE F2)
p45: CCCATTCTCCTCRACRATTCARC (BS AmpE R1.2)
p46 TGACATGGATTCTTCGAGGCCT (ETS TAQMAN for)
p47 CATGACACGCCCATTCTCTTCG (ETS taqman Rev)
p48 TATATAACTTGTTCGCATGATATT (6FAM)
p49 TATATTGGGAAAGCGCATGGT (VIC)
mir159: tagagctcccttcaatccaaa
AtSIN1 E12Si1: aagcttagaggtcgccggttcgagtaatgcttggaacgaaact
siRNA 45S: gtctgttggtgccaagagggaaaagggctaat
snRNA U6: aggggccatgctaatcttctc
U3 3AtU3: CTGTCAGACCGCCGTGCGA
5'eIF1a: ctaaggatggtcagacccg
3'eIFa: cttcaggtatgaagacacc
Act-For: GAGAGATTCAGATGCCCAGAAGTC 
Act-Rev: TGGATTCCAGCAGCTTCCA

Supporting figure legends

Figure S1: Similar growth and molecular phenotypes of two independent Atnuc-L1 mutant plants A) Top, picture of WT, Atnuc-L1-1 and Atnuc-L1-2 plants grown on soil ~4 weeks under 16:8 –h (light: dark) cycle. The bar graph shows shoot fresh weight (in grams per plant) from WT and Atnuc-L1-1 and Atnuc-L1-2 plants. Bottom, diagram of the AtNUC-L1 gene from the ATG start to the TGA stop codon. The black boxes correspond to exons separated by 14 introns. The T-DNA insertion in the Atnuc-L1-1 (salk_053590) and Atnuc-L1-2 (salk_002764) plants is indicated. B) Top, PCR reactions using cDNA prepared from RNA isolated from WT (lane 1) and Atnuc-L1 (lanes 2 and 3) plants to detect AtNUC-L1 transcripts. eIF1á gene expression was analyzed to evaluate the amount of cDNA used in each reaction. Bottom, the level of AtNUC-L2 transcripts in both Atnuc-L1 mutant plants was determined by qPCR. C) Western blot analysis using specific antibodies against the AtNUC-L1 and AtNUC-L2 peptides and total protein extracts from WT and Atnuc-L1 mutant plants. The diagram shows the sequence and position of the peptide in the C-terminal region of protein AtNUC-L1. See [17] for PCR and western blot conditions. 

Figure S2. Complementation studies. A) Top, plant growth and development defects in mutant plants are fully restored in transformed Atnuc-L1 mutant plants: a, WT; b and c, Atnuc-L1-1 Atnuc-L1-2, d and e transformed Atnuc-L1-1 Atnuc-L1-2 plants. Bottom, Atnuc-L1 mutant plants were transformed with AtNUC-L1 genomic sequences fused to epitope tag flag-HA. Genomic DNA includes 1.1 kb sequence upstream from staring ATG and AtNUC-L1 coding region (exons and introns minus TGA stop codon).B) RT PCR reaction to detect rRNA variants (3'ETS rRNA panel) in WT (lane 1), Atnuc-L1 (lanes 2 and 3) and complemented plants (lanes 4 and 5). Amplification of eIF1á mRNA was used to controls total amount of RNA in each sample (Panel eIF1á). Amplification of eIF1á genomic DNA shows absence of DNA contamination in the RNA samples (lane 6). C) Western blot analysis using specific antibodies against the AtNUC-L1 peptide and total protein extract from WT (lane 1), Atnuc-L1 (lanes 2 and 3) and transformed plants (lane 4 and 5). The same membrane was hybridized with antibodies against a NADPH Thioredoxine Reductase (NTR) to verify similar amount of protein in each sample.

Figure S3. Alignment of 3'ETS rDNA sequences cloned either by PCR using genomic DNA (VAR1, VAR 2 and VAR3) or by RT-PCR using total RNA (VAR4) from WT plants. The grey solid line shows the 3'end of the 25S rRNA sequence and the black solid lines repeat (R1-R4) sequences located downstream of 3'ETS cleavage site detected by RNase mapping. The double line just upstream of R2, shows the position of the triplet CAC missed in the VAR2a and VAR3a isoforms (not shown). Primers used to detect specific rRNA variants (p32/p33 and p1/p5 for VAR1 and VAR4 respectively) and all rRNA variants (p3/p4) are shown by dotted arrows.

Figure S4: Processing of accumulated pre-rRNA in Atnuc-L1 mutant plants is accurate. A) Northern blot analysis using total RNA isolated from WT and Atnuc-L1 mutant plants and [ã32P] 5'-end labeled primers (p34-p41) to detect 5'ETS1 (p34, lanes 1-3), 5'ETS2 (p35, lanes 4-6), 18S (p36, lanes 7-9), ITS1 (p37, lanes 10-12), 5.8S (p38, lanes 13-15), ITS2 (p39, lanes 16-18), 25S (p40, lanes 19-21) and 3'ETS (p41, lanes 22-24) pre-rRNA sequences. B) The agarose gel stained with GelRed (BioTium) show similar amounts of total RNA in WT (lane 3) and Atnuc-L1 (lanes 1 and 2) RNA samples. C) The diagram represents a model of pre-rRNA processing steps and rRNA mature and intermediates detected both in WT and Atnuc-L1 mutant plants. The smear detected with p34 might correspond to an exonucleolytic trimming of the 5'end product from 5'ETS cleavage in the P site, while the signal indicated by asterisk the an rRNA intermediates generated by an alternative cleavage upstream of P site (depicted by a vertical arrow). Panels 5ETS1, 5ETS2 18S and 3ETS (shown in figure 4) are presented here to pinpoint position of intermediated and mature rRNA.

Figure S5. Accumulation of RNA Pol I sub unit and pre-rRNA in Atnuc-L1 mutant plants. Left, in situ hybridization of pre-rRNA in nucleoli of roots from WT and Atnuc-L1-1. Hybridization was performed using a pre- rRNA probe (shown in the Fig.) to detect 5'ETS uncleaved pre-rRNA. Right, Inmunolocalization of RNA polI subunit in WT and Atnuc-L1-1 plants using antibodies against the 24.3 kDa subunit. Bar=10 ìm. In Panel pre-rRNA, the accumulated small punctuate loci dispersed throughout the nucleolus of Atnuc-L1 plants can be compared with the multiple transcriptional initiation sites detected by BrU incorporation in pea and other species [6]. In Panel á-24.3, we observed a localization of the 24.3 kDa subunit in “focus” both in the nucleolus of WT and AtnucL1 plant mutants (Panel á-PolI). Although in the nucleolus of Atnucl-L1 plants these “foci” are less numerous they are larger in size compared with those observed in the nucleolus of WT plants. 

Figure S6: AtNUC-L1 gene disruption does not affect histone epigenetic marks. A. Chromatin samples prepared from WT (light grey bars), Atnuc-L1-1 (dark grey bars) and Atnuc-L1-2 (black bars) plants were inmunoprecipitated with antibodies against H3Ac, H3K4me2 and H3K9me2. ChIP samples were then amplified with specific primers to 5'ETS (p12/p13), 25S (p14/p15), 3'ETS (p16/p17) and IGS2 (p18/p19) rRNA gene sequences. Amplification of ACT2 and soloLTR gene was performed to control ChiP and qPCR reactions. 

Figure S7: rRNA VAR1 gene expression in Atnuc-L1-1 mutant plants. RT PCR reaction was performed with total RNA prepared from individual WT (lanes 1-5) and Atnuc-L1-1 (lanes 6-10) mutant plants and using primers p3-p4 to detect 3'ETS rRNA variant expression. Amplification of eIF1á mRNA was used to controls total amount of RNA in each sample (Panel eIF1á). Amplification of eIF1á genomic DNA shows absence of DNA contamination in the RNA samples (lane 11).

Figure S8: AtNUC-L1 binds 5'ETS but it does not interacts with RNA Pol I. A) Analysis of 5'ETS binding activity on 4% polyacrylamide gel. EMSA was performed with 1, 5, 10 and 15 ìl (lanes 2-5) of recombinant His-AtNUC-L1 protein (0.6 ìg/ml) protein and with a á-32P-dCTP filling Klenow labeled rDNA probe. Lane 1, rDNA probe alone B) Inmunoprecipitation (IP) was performed with total protein extracts and antibodies against AtNUC-L1 coupled to agarose beads. After IP reaction total (lane1), unbound (lane 2) and bound (lane 3) proteins were subjected to PAGE and analyzed by Western blot using antibodies against AtNUC-L1, the 24.3 kDa subunit of RNA Pol I and fibrillarin from Arabidopsis (AtFib). C) Diagram of the rDNA probe A123B used in the EMSA assay. The probe encompasses genomic 5'ETS rDNA nucleotides from +96 to +327. 

Figure S9 - AtNUC-L1 binds actives rRNA genes. Chromatin samples prepared from the allotetraploid Arabidopsis suecica (where the thaliana derived rRNA genes are repressed and those of arenosa are expressed) were inmunoprecipitated with antibodies against AtNUC-L1. The 5'ETS sequences were detected by PCR using primers p46/p47 and labeled primers p48 or p49 specific to 5'ETS sequences from A. thaliana or A. arenose respectively. Amplification of ACT2 gene was performed to control ChiP and qPCR reactions. The qPCR reactions were performed using the TaqMan system (Premier Biosoft).

Figure S10 - pre-rRNA transcripts levels in backcrossed F1 WT x Atnuc-L1-2 plants. RT PCR reaction to detect pre- rRNA transcripts in WT (lane 1), Atnuc-L1-2 (lanes 2) and F1 WT x Atnuc-L1-2 (lane 3) plants Amplification was performed with primers p3/p4 to detect 3'ETS rRNA variants (Panel 3'ETS VAR), primers p7/p8 and p42/p43 to detect transcripts from IGS (Panels -223/+243 and -507/-201 respectively). Amplification of actin mRNA (Panel ACT2) was used to control total amounts of RNA in each sample. No amplification bands were detected in RT-PCR reactions without reverse transcriptase

Figure S11. Bisulfite sequencing analysis. The bar graphs show the representation (%) of methylated sites in the rRNA gene promoter sequences (from -315 to +1) from WT and Atnuc-L1 mutant plants in a CG (upper panel), CHG (middle panel) and CHH (lower panel) context. The schema with the position of primers p44/p45 (located at -315 and at +243) used for sequencing bisulfite treated samples is shown in figure 7.
